# Vitamin D Inhibits IL-22 Production Through a Repressive Vitamin D Response Element in the *il22* Promoter

**DOI:** 10.3389/fimmu.2021.715059

**Published:** 2021-08-02

**Authors:** Daniel V. Lopez, Fatima A.H. Al-Jaberi, Nkerorema D. Damas, Brian T. Weinert, Urska Pus, Sara Torres-Rusillo, Anders Woetmann, Niels Ødum, Charlotte M. Bonefeld, Martin Kongsbak-Wismann, Carsten Geisler

**Affiliations:** ^1^The LEO Foundation Skin Immunology Research Center, Department of Immunology and Microbiology, Faculty of Health and Medical Sciences, University of Copenhagen, Copenhagen, Denmark; ^2^The Novo Nordisk Foundation Center for Biosustainability, Technical University of Denmark, Kgs. Lyngby, Denmark

**Keywords:** Th22 cells, IL-22, IL-17, vitamin D, vitamin D receptor, vitamin D response element (VDRE)

## Abstract

Th22 cells constitute a recently described CD4^+^ T cell subset defined by its production of interleukin (IL)-22. The action of IL-22 is mainly restricted to epithelial cells. IL-22 enhances keratinocyte proliferation but inhibits their differentiation and maturation. Dysregulated IL-22 production has been associated to some inflammatory skin diseases such as atopic dermatitis and psoriasis. How IL-22 production is regulated in human T cells is not fully known. In the present study, we identified conditions to generate Th22 cells that do not co-produce IL-17 from naïve human CD4^+^ T cells. We show that in addition to the transcription factors AhR and RORγt, the active form of vitamin D_3_ (1,25(OH)_2_D_3_) regulates IL-22 production in these cells. By studying T cells with a mutated vitamin D receptor (VDR), we demonstrate that the 1,25(OH)_2_D_3_-induced inhibition of *il22* gene transcription is dependent on the transcriptional activity of the VDR in the T cells. Finally, we identified a vitamin D response element (VDRE) in the *il22* promoter and demonstrate that 1,25(OH)_2_D_3_-VDR directly inhibits IL-22 production *via* this repressive VDRE.

## Introduction

Various subsets of effector CD4^+^ T helper (Th) cells, classified by the lineage-specific master transcription factors they express and the cytokines they secrete, have been described (1, 2). Th22 cells constitute a recently described CD4^+^ T cell subset defined by their production of interleukin (IL)-22 (3, 4). The biological functions of IL-22 is mainly restricted to non-hematopoietic cells such as epithelial cells located in the skin, gut, lung, liver, pancreas and kidney (5–7). In the skin and gut epithelium, IL-22 induces secretion of several anti-microbial peptides that contribute to the defence mechanisms against microorganisms (8–10). Furthermore, IL-22 enhances proliferation of keratinocytes while inhibiting their differentiation and maturation, implying an important role of IL-22 in the homeostasis of the skin (11–13). The role of IL-22 in epithelial homeostasis is further underlined by its association to inflammatory skin and gut diseases such as atopic dermatitis (14, 15), psoriasis (16) and colorectal cancers (17). Th22 cells are closely related to Th17 cells, and Th17/Th22 cells co-producing IL-17A and IL-22 have been described (10, 18, 19). However, distinct IL-22-producing Th22 cells that do not produce IL-17 have been isolated from both humans (3, 4, 20) and mice (21).

Other types of immune cells than Th22 cells, such as innate lymphoid cells (ILC), γδ T cells and natural killer (NK) cells can produce IL-22 (22–24). It has been found that the transcription factor RORγt plays an important role in the regulation of IL-22 secretion in human and mouse ILC3 (25–28). Furthermore, IL-21 and the aryl hydrocarbon receptor (AhR) play regulatory roles in IL-22 production in mouse CD4^+^ T cells (29). However, the transcription factors involved in IL-22 regulation in human Th22 cells are still not fully known. One study has found that both RORγt and AhR are important for Th22 differentiation and IL-22 production (20), whereas another study found that RORγt is undetectable in Th22 cells (4).

The active form of vitamin D_3_, 1,25(OH)_2_D_3_, modulates the expression of many genes *via* binding to the intracellular vitamin D receptor (VDR) (30, 31). Ligand-bound VDR form heterodimers with retinoid X receptors (RXR) and translocate to the nucleus (30, 31). Here, 1,25(OH)_2_D_3_-VDR : RXR heterodimers act as transcription factors by binding to vitamin D response elements (VDRE) located in the regulatory regions of vitamin D-regulated genes (30–34). Binding of 1,25(OH)_2_D_3_-VDR : RXR heterodimers to VDRE leads to either activation or repression of target gene transcription (32). Interestingly, 1,25(OH)_2_D_3_ regulates the differentiation of CD4^+^ T cells. Thus, 1,25(OH)_2_D_3_ promotes differentiation of Th2 cells by enhancing IL-4 production and concomitantly represses Th1 differentiation by repressing interferon (IFN) γ production. Moreover, 1,25(OH)_2_D_3_ promotes the differentiation of regulatory T (Treg) cells and inhibits Th17 cell differentiation (35–41). The effect of 1,25(OH)_2_D_3_ on human Th22 cell differentiation and IL-22 secretion is not fully known. Some studies have suggested that 1,25(OH)_2_D_3_ promotes the differentiation of Th22 cells (4, 42), whereas other found that 1,25(OH)_2_D_3_ inhibits IL-22 production (43).

The aim of this study was to determine how 1,25(OH)_2_D_3_ controls IL-22 production in human T cells.

First, we established the conditions for *in vitro* differentiation of human naïve CD4^+^ T cells to Th22 cells. We found that activation of naïve CD4^+^ T cells with allogeneic dendritic cells (DC) in the presence of TNFα, IL-6, IL-23, IL-1β, the AhR agonist FICZ and the transforming growth factor-β (TGFβ) receptor type 1 inhibitor galunisertib led to optimal generation of Th22 cells. We confirmed that the transcription factors AhR and RORγt regulate IL-22 in these cells. Importantly, we found that 1,25(OH)_2_D_3_ inhibits IL-22 production in human Th22 cells. We show that 1,25(OH)_2_D_3_-mediated inhibition of IL-22 was not due to inhibition of AhR, RORγt or STAT-3. In contrast, we identified a novel VDRE in the *il22* promoter by which 1,25(OH)_2_D_3_-VDR : RXR complexes directly represses IL-22 transcription.

## Materials and Methods

### Reagents and Chemicals

TNFα (210-TA), IL-1β (201-LB), IL-6 (206-IL) and IL-23 (1290-IL) were purchased from R&D systems. Galunisertib (LY2157299) was purchased from Selleckchem. 1,25(OH)_2_D_3_ (BML-DM200-0050) were from, Enzo Life Sciences, Inc., Ann Arbor, MI. Stock 1,25(OH)_2_D_3_ solution of 2.4 mM were diluted in >99.5% ethanol anhydrous. AhR agonist (FICZ, 5304) and AhR antagonist (CH-223191, 3858) were from TOCRIS Inc. FICZ and CH-223191 were solubilized in DMSO to make a 25 mM and a 100 mM stock solution, respectively. RORγt antagonist (SR-2211) was from TOCRIS Inc. SR-2211 (4869) was solubilized in DMSO to make a 10 mM stock solution. Recombinant human IL-21 (200-21) was from PeproTech and anti-IL-21 (NBP1-76740) was from Novus Biologicals.

### T Cell Isolation and Activation

All procedures involving the handling of human samples were in accordance with the principles described in the Declaration of Helsinki and the samples were collected and analysed according to ethically approval by the Regional Ethical Committee of the Capital Region of Denmark (H-16033682). Peripheral blood mononuclear cells (PBMC) were purified from healthy donor’s blood by density gradient centrifugation using Lymphoprep (Axis-Shield, Oslo, Norway). Subsequently, naive CD4^+^ T cells were isolated by negative selection using Easysep Human Naive CD4^+^ T cell Enrichment Kit (19155 Stemcell Technologies) according to the manufacturer’s protocol. In short, PBMC were incubated with antibodies targeting undesired cells, and subsequently magnetic particles were used to bind undesired cells. Hereafter, these cells were retained using an EasySep Magnet (18000, Stemcell Technologies). The resulting cell population consisted of >95% naïve CD4^+^ T cells. The obtained cells were cultured at a concentration of 1 x 10^6^ cells/ml serum-free X-VIVO 15 medium (BE02-060F, Lonza, Verviers, Belgium), and activated with allogeneic dendritic cells (DC) in a 1:10 DC:T cell ratio or activated with Dynabeads Human T-activator CD3/CD28 (111.31D, Life Technologies, Grand Island, NY) in a 2:5 bead:T cell ratio in flat-bottomed 24 well culture plates (142475, Nunc). T cells were activated for four days at 37°C, 5% CO2 under polarizing conditions for Th0 cells (un-supplemented X-VIVO 15 medium) and Th22 cells (X-VIVO 15 medium supplemented with TNF (10 ng/ml), IL-1β (10 ng/ml), IL-6 (30 ng/ml), IL-23 (20 ng/ml), FICZ (0.3 µM) and galunisertib (10 µM)). In some experiments CH-223191, SR-2211, 1,25(OH)_2_D_3_, anti-IL-21 and recombinant human IL-21 were added at the indicated concentrations to the medium during the activation period. For kinetic experiments, naïve CD4^+^ T cells were cultured in flat-bottomed 24-well culture plates in X-VIVO 15 medium and activated with beads or with allogeneic dendritic cells as described above in the presence of Th22 polarizing conditions for 0-144 h.

### Dendritic Cell Differentiation

Dendritic cells (DC) were differentiated from isolated human monocytes. Human monocytes were purified from PBMC using Easysep Human Monocyte Enrichment Kit (19059, Stemcell Technologies) according to the manufacturer’s protocol. 1.5 x 10^6^ monocytes were cultured in flat-bottomed 6-well culture plates (140675, Nunc) for six days in 3 ml DC medium (RPMI-1640 medium (R5886, Sigma Aldrich) supplemented with 1% Penicillin/Streptomycin, 1% L-Glutamine and 10% heat-inactivated and endotoxin-free fetal bovine serum (FBS) (10082-147, Gibco)) in the presence of GM-CSF and IL-4 (both 50 ng/ml, AF-HDC, Peprotech). After three days, fresh DC medium was added. On day five, differentiated DC were supplemented with GM-CSF (50 ng/ml) and treated with heat-killed mycobacterium Tuberculosis (HKMT) (10 ng/ml) (tlrl-hkmt, InvivoGen) for 24 h. Activated DC were washed with PBS and resuspended in X-VIVO 15 for mono- and co-cultures. For mono-culture, 5 x 10^5^ DC/ml were cultured in flat-bottomed, 24-well plates and activated for 0-120 h with the indicated concentration of HKMT under Th22 polarizing conditions. For co-culture experiments, 1 x 10^6^ naïve human CD4^+^ T cells were co-cultured with 1 x 10^5^ allogeneic DC per ml in flat-bottomed, 24-well culture plates in X-VIVO 15 medium under Th22 polarizing conditions.

### Cell Lines

The malignant T cell line MyLa 2059 was previously established from a plaque biopsy specimen of a patient with cutaneous T cell lymphoma (CTCL) (44). 5 x 10^5^ cells/ml were cultured in flat-bottomed, 24-well plates in RPMI-1640 supplemented with 1% Penicillin/Streptomycin, 1% L-Glutamin and 10% heat-inactivated and endotoxin-free FBS in the absence or presence of Th22 polarizing conditions and the indicated concentrations of 1,25(OH)_2_D_3_ for 48 h.

### Antibodies and Flow Cytometry

Anti-CD4 BV711 (SK3), anti-CD80 BV605 (L307), anti-CD25 PE-Cy7 (M-A251), anti-CD38 BV421 (HIT2) were purchased from BD Biosciences (Franklin Lakes, NJ). Fixable viability dye (efluor 780) was purchased from eBioscience (San Diego, CA). Anti-IL-17 APC (EBIO64DEC17) and anti-IL-22 PE (22URTI) were purchased from ThermoFisher Scientific (Life Technologies Europe BV, Roskilde, DK). Anti-IgG1k APC (QA16A12) was purchased from Biolegend (San Diego, CA) and anti-IgG1k PE (MOPC-21) was purchased from BD Biosciences (Franklin Lakes, NJ). For analyses of intracellular cytokines, cells were re-stimulated with PMA (1 µg/ml) (P8139, Sigma), ionomycin (1 µg/ml) (I0634, Sigma) in the presence of monensin (2 µg/ml) (M5273, Sigma) for 4 h at 37°C, 5% CO2. The cells were stained for surface markers, fixed and permeabilized with the Fixation/Permeabilization Solution Kit (BD Bioscience) and subsequently stained cytokine-specific antibodies. The cells were analysed on a Fortessa 5 laser flow cytometer using FACSDiva software and further analysed using FlowJo software. Neutralizing anti-IL-21 antibodies (NBP1-76740) were from Novus Biologicals.

### RT-qPCR

mRNA levels for various targets were measured by RT-qPCR. Following cell isolation, cells were lysed in TRI reagent (T9424, Sigma Aldrich) and mixed with phase separation reagent 1-bromo-3-chloropropane (B9673, Sigma Aldrich). The RNA phase was isolated and mixed with isopropanol supplemented with glycogen for RNA precipitation (10814-010, Invitrogen). The RNA pellet was then washed in RNase free 75% ethanol 3 times. cDNA was synthesized from quantified RNA using High-Capacity RNA-to-cDNA™ Kit (4387406, Applied Biosystems) according to manufacturer’s instructions. For RT-qPCR, 12.5 ng of cDNA was mixed with TaqMan^®^ Universal Master Mix II with UNG (4440038, Applied the target primer and RNase and DNase free water for normalization. The following target primers were used: IL-22 (Hs01574154_m1), STAT-3 (Hs01047580_m1), AhR (Hs00907314_m1), RORc (Hs01076122_m1), IL-21 (Hs00222327_m1), GAPDH (Hs99999905_m1). The plate-based detection instrument LightCycler ^®^ 480 II from Roche was used for real-time PCR amplification.

### Cytokine Measurements

IL-17A and IL-22 in the supernatant were measured by ELISA according to the manufacturer´s instruction (InVitroGen, IL-17A 88-7176-22 and IL-22 88-7522-88).

### Western Blotting Analysis

For Western blotting analysis, cells were lysed with lysis buffer containing 50 mM Tris-base, 150 mM NaCl, 1 mM MgCl_2_ supplemented with 1% (v/v) Triton X-100, 1 x Protease/Phosphatase Inhibitor Cocktail (5872S, Cell Signalling Technology) and 5 mM EDTA. The lysates were vortexed for 5 seconds every 5 minutes for 25 minutes at room temperature and subsequently centrifuged at 10.000 G for 10 minutes at 4°C. Loading buffer containing lithium dodecyl sulphate (LDS) (NP0007, Life Technologies) along with reducing agent (NP0009, Life Technologies) was added and the lysates separated by electrophoresis through NuPAGE™ 10% BisTris gels (NP0302BOX or NP0301BOX, Life Technologies). The proteins were transferred to nitrocelulose membranes (LC2001, Life Technologies). The membranes were blocked in 5% skim milk dissolved in Tris-buffered saline with 0,1% tween (TBST), washed 3 times in TBST for 3 minutes and incubated overnight at 4°C with target-specific primary antibodies (anti-STAT-3 (D1B2J) and anti-phospho-STAT3 (9145) from Cell Signaling Technology, anti-VDR (D-6), anti-AhR (sc-5579), anti-RORγt (sc-293150) and anti-GAPDH (sc-365062) from Santa Cruz Biotechnology) diluted in TBST supplemented with 5% BSA. The membranes were subsequently washed and incubated with a secondary antibody (swine anti-rabbit Ig or rabbit anti-mouse Ig (P0399 and P0260 from Dako, Glostrup, Denmark S/A), conjugated with horseradish peroxidase (HRP) and diluted in 5% skim milk. Finally, membranes were washed and exposed to ECL luminescence reagent (RPN2232, Sigma Aldrich). The corresponding signals were detected using a ChemiDocTM MP Imaging System (Bio Rad) and the software ImageLab.

### Plasmids

To investigate the presence of VDRE in the *il22* promoter, bioinformatics analysis of the *il22* gene (HGNC : HGNC:14900) was performed using JASPAR, an open-access database of transcription factor binding profiles, where several combinations of the VDR-RXR complex binding profile on several genes are described (45). The cloning of the *il22* promoter into the pMCS Tluc16 Hygro Vector expressing luciferase (88255 from ThermoFisher Scientific) was performed by using the restriction enzymes *KpnI* and *Hind III via* Invitrogen GeneArt Gene Synthesis. The generated plasmid construct contained the *il22* promoter, including the putative VDRE located 2159-2173 bases upstream from the start codon, driving the expression of Tluc16 luciferase gene (IL-22-Tluc VDRE-WT) and having the antibiotic resistance genes ampicillin (AmpR) and hygromycin (HygroR).

### Directed Mutagenesis

The generation of the construct IL-22-Tluc with deletion of the VDRE (IL-22-Tluc VDRE-KO) was performed using the GeneArt™ Site-Directed Mutagenesis System (A13282, ThermoFisher Scientific) according to the manufacturer’s protocol for long plasmids ~10 Kb. The following primers were designed and used to remove the VDRE:

Forward primer: 5’-ATTCCTTCTAATTGTATCGTACCTCTCCCCATCCTCCT-3’
Reverse primer: 5’-AGGAGGATGGGGAGAGGTACGATACAATTAGAAGGAAT -3’

### Luciferase Assay

Nucleofection of 5 x 10^5^ Myla 2059 cells/well with 200 ng of the constructs IL-22-Tluc VDRE-WT and IL-22 VDRE-KO were performed using the P3 Primary Cell 96-well Nucleofector™ Kit (V4SP-3096) and program EH-140 on the Lonza Nucleofector 96-well Shuttle (LZ-AAM-1001S). Subsequently, 5 x 10^5^ electroporated cells/ml were cultured in flat-bottomed 24-well plates in RPMI-1640 with 1% L-glutamine and 10% heat-inactivated and endotoxin-free fetal bovine serum in the presence of the indicated concentrations of 1,25(OH)_2_D_3_ at 37°C in 5% CO_2_. After 48 h of incubation, luciferase activity was measured as counts per second (CPS) using the TurboLuc™ Luciferase One-Step Glow Assay Kit (88263) according to the manufacturer’s protocol.

### Statistical Analysis

Two-tailed, paired Student’s t-tests were used to compare responses in the same group of cells treated in two different ways. Significance levels are indicated as follows: * p < 0.05; ** p < 0.01; *** p < 0.005; **** p < 0.001. Data are presented as mean values with one standard error of the mean (SEM). The number of donors as well as the number of independent experiments are indicated in the figure legends.

## Results

### Differentiation of Human Th22 Cells *In Vitro*

**Figure 1:**
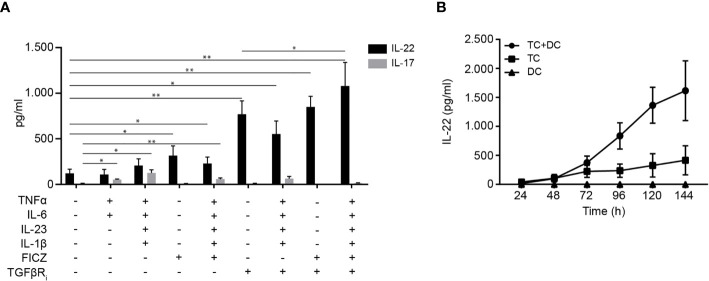
Differentiation of human Th22 cells *in vitro.* **(A)** IL-22 and IL-17 in the supernatant of naïve CD4^+^ T cells co-cultured with allogeneic DC for 96 h in the absence or presence of TNFα (10 ng/ml), IL-1β (10 ng/ml), IL-6 (30 ng/ml) IL-23 (20 ng/ml), FICZ (0.3 µM) and TGFβR inhibitor (galunisertib,10 µM), (mean + SEM, two independent experiments with 5 donors). **(B)** IL-22 in the supernatant of DC-T cell co-cultures, T cell mono-cultures activated with CD3/CD28 beads and DC mono-cultures after 24-144 h of culture in Th22 medium (mean ± SEM, two independent experiments with 4 donors).

Presently, there is no consensus on the conditions required for *in vitro* generation of human Th22 cells. One study has identified IL-1β and IL-23 as the optimal cytokine cocktail to generate Th22 cells that do not produce IL-17 (20), whereas another study found that tumour necrosis factor (TNF) and IL-6 were required for optimal Th22 cell generation (4). To establish conditions for efficient differentiation of human CD4^+^ T cells to Th22 cells in a physiological-like setting, we activated naïve CD4^+^ T cells with allogeneic DC and investigated the combinatory effect of several factors believed to induce IL-22 transcription. After 96 h of culture, we measured IL-22 and IL-17 in the supernatant. In our hands, neither the IL-1β/IL-23 nor the TNF/IL-6 combination significantly increased IL-22 production compared to untreated cells (**Figure 1A**). The combination of TNF, IL-1β, IL-6, and IL-23 induced both IL-22 and IL-17 secretion (**Figure 1A**). The AhR agonist FICZ and the TGF-βR inhibitor galunisertib markedly increased IL-22 secretion without stimulating IL-17 secretion. Although modestly, addition of FICZ and the cytokines to galunisertib significantly increased the secretion of IL-22 without increasing the secretion of IL-17. Thus, in our hands medium supplemented with TNF, IL-1β, IL-6, IL-23, FICZ and galunisertib (from here on termed Th22 medium) resulted in optimal differentiation of human naïve CD4^+^ T cells towards Th22 cells that secreted high levels of IL-22 and no IL-17 (**Figure 1A**).

Next, we wanted to characterize differentiation of naïve CD4^+^ T cells in mono-cultures versus in co-cultures with allogeneic DC and furthermore to determine the time required for efficient differentiation to Th22 cells and secretion of IL-22. To do this, we measured the IL-22 concentration at day 1-6 in the supernatants of allogeneic DC-T cell co-cultures, of mono-cultures of CD4^+^ T cells activated with CD3/CD28 beads and of mono-cultures of activated DC all in Th22 medium. We found that the allogeneic DC-T cell co-cultures produced significantly more IL-22 than T cells activated with CD3/CD28 beads in mono-culture (**Figure 1B**). Moreover, we observed that DC in mono-culture did not secrete IL-22, underlining that IL-22 is produced by T cells. Furthermore, we found that IL-22 production reached maximum and plateaued out at 96 h in the DC-T cell co-cultures (**Figure 1B**). Consequently, we chose DC-T cell co-cultures incubated for 96 h for the following Th22 differentiation experiments.

### AhR and RORγt Regulate IL-22 Production in Human Th22 Cells

**Figure 2:**
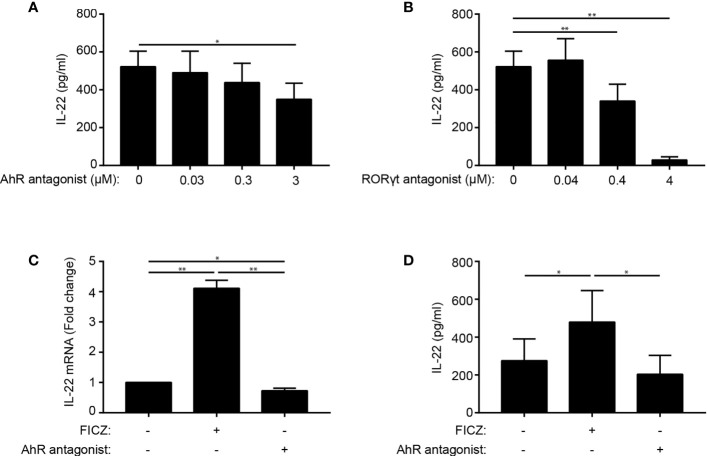
AhR and RORγt regulate IL-22 production in human Th22 cells. IL-22 in the supernatant of naïve CD4^+^ T cells co-cultured with allogeneic DC for 96 h in Th22 medium and the presence of **(A)** the AhR antagonist CH-223191 or **(B)** the RORγt antagonist SR-2211 in the indicated concentrations. (mean + SEM, n = 4). **(C)** Relative mRNA expression and **(D)** IL-22 production in DC-T cell co-cultures in Th22 medium in the absence or presence of FICZ (0.3 µM) or CH-223191 (3 µM). For mRNA expression, the data are normalized to the values obtained from DC-TC co-cultures in Th22 medium in the absence of FICZ and CH-223191 (mean + SEM, one experiment with 4 donors).

Conflicting data on the role of AhR and RORγt in IL-22 production in human Th22 cells have been presented (4, 20). To determine the role of these transcription factors, we stimulated naïve CD4^+^ T cells with allogeneic DC in Th22 medium and increasing concentrations of the AhR antagonist CH-223191 or the RORγt antagonist SR-2211. After 96 h of culture, we measured IL-22 in the supernatant. We observed that both the AhR and the RORγt antagonist down-regulated IL-22 production (**Figures 2A, B**). The AhR and RORγt antagonists did not affect T cell activation or viability in the concentrations used in the present study (**Supplementary Figure 2**). To further explore the regulatory role of AhR on IL-22 production, we activated naïve CD4^+^ T cells in Th22 medium in the absence or presence of the AhR agonist FICZ and the AhR antagonist CH-223191. We found that the AhR agonist up-regulated and the AhR antagonist down-regulated IL-22 mRNA and IL-22 secretion (**Figures 2C, D**). Taken together, these data indicate that AhR and RORγt play key roles in the regulation of IL-22 in human Th22 cells.

### 1,25(OH)_2_D_3_ Inhibits IL-22 Production in Human Th22 Cells

**Figure 3:**
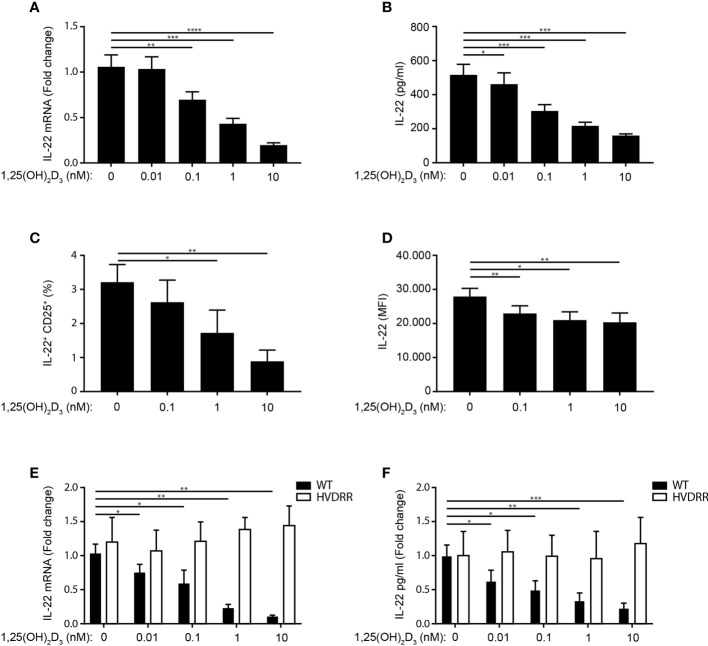
1,25(OH)_2_D_3_ inhibits IL-22 production in human Th22 cells. **(A)** Relative mRNA expression **(B)** and production of IL-22 in naïve CD4^+^ T cells co-cultured with allogeneic DC for 96 h in Th22 medium and the indicated concentrations of 1,25(OH)_2_D_3_. The data in **(A)** are normalized to the values obtained from DC-TC co-cultures incubated in Th22 medium in the absence of 1,25(OH)_2_D_3_ (mean + SEM, six independent experiments with 10 donors). **(C)** Frequency of IL-22^+^ CD25^+^CD4^+^ T cells after activation of naïve CD4^+^ T cells with allogeneic DC for 96 h in Th22 medium and the indicated concentrations of 1,25(OH)_2_D_3_ (mean percentage of positive cells + SEM, three independent experiments with 5 donors). **(D)** Mean fluorescent intensity (MFI) of IL-22 in the IL22^+^CD25^+^ T cells described above (mean expression of IL-22 + SEM, three independent experiments with 5 donors). **(E)** Relative mRNA expression and **(F)** IL-22 production in naïve CD4^+^ T cells from healthy individuals (black) and from the HVDRR patient (white) activated with allogeneic DC for 96 h in Th22 medium and the indicated concentrations of 1,25(OH)_2_D_3_. The data are normalized to the values obtained from CD4^+^ T cells activated in the absence of 1,25(OH)_2_D_3_ (mean + SEM, two independent experiments with 4 donors).

Contradictory data on the effect of 1,25(OH)_2_D_3_ on IL-22 production in human Th22 cells have been published (4, 42, 43). To determine the effect of 1,25(OH)_2_D_3_ in Th22 cells, we activated naïve CD4^+^ T cells with allogeneic DC in Th22 medium in the absence or presence of 1,25(OH)_2_D_3_. After 96 h of culture, we subsequently measured IL-22 mRNA expression levels in the cells and IL-22 in the supernatant. We found that 1,25(OH)_2_D_3_ inhibited IL-22 mRNA expression and IL-22 secretion (**Figures 3A, B**). In accordance, the frequency of IL-22^+^ activated CD4^+^ T cells and their IL-22 mean fluorescent intensity (MFI) were down-regulated by 1,25(OH)_2_D_3_ (**Figures 3C, D**, for gating strategy please see **Supplementary Figure 1A**). We found that Th22 express the VDR and that 1,25(OH)_2_D_3_ inhibits IL-22 production in Th22 mono-cultures, supporting a direct inhibitory effect of 1,25(OH)_2_D_3_ on the Th22 cells (**Supplementary Figure 3**). Furthermore, we found that 1,25(OH)_2_D_3_ did not affect T cell activation or viability in the concentrations used in the present study (**Supplementary Figure 5**).

To establish that the inhibitory effect of 1,25(OH)_2_D_3_ on IL-22 expression and production was mediated *via* the VDR, we determined the effect of 1,25(OH)_2_D_3_ on IL-22 in parallel in CD4^+^ T cells from controls and from a patient with hereditary vitamin D resistant rickets. This patient has a mutation in the DNA-binding domain of the VDR that abolishes the transcriptional activity of the VDR (46). Whereas 1,25(OH)_2_D_3_ clearly down-regulated IL-22 expression and production in control T cells, it had no effect on IL-22 in T cells from the patient (**Figures 3E, F**). Taken together, these data demonstrated that 1,25(OH)_2_D_3_ inhibits IL-22 expression and secretion in human Th22 cells and that the 1,25(OH)_2_D_3_-induced inhibition of IL-22 is dependent on the transcriptional activity of the VDR.

### 1,25(OH)_2_D_3_ Does Not Inhibit IL-22 by Affecting AhR, RORγt or STAT-3 Expression

**Figure 4:**
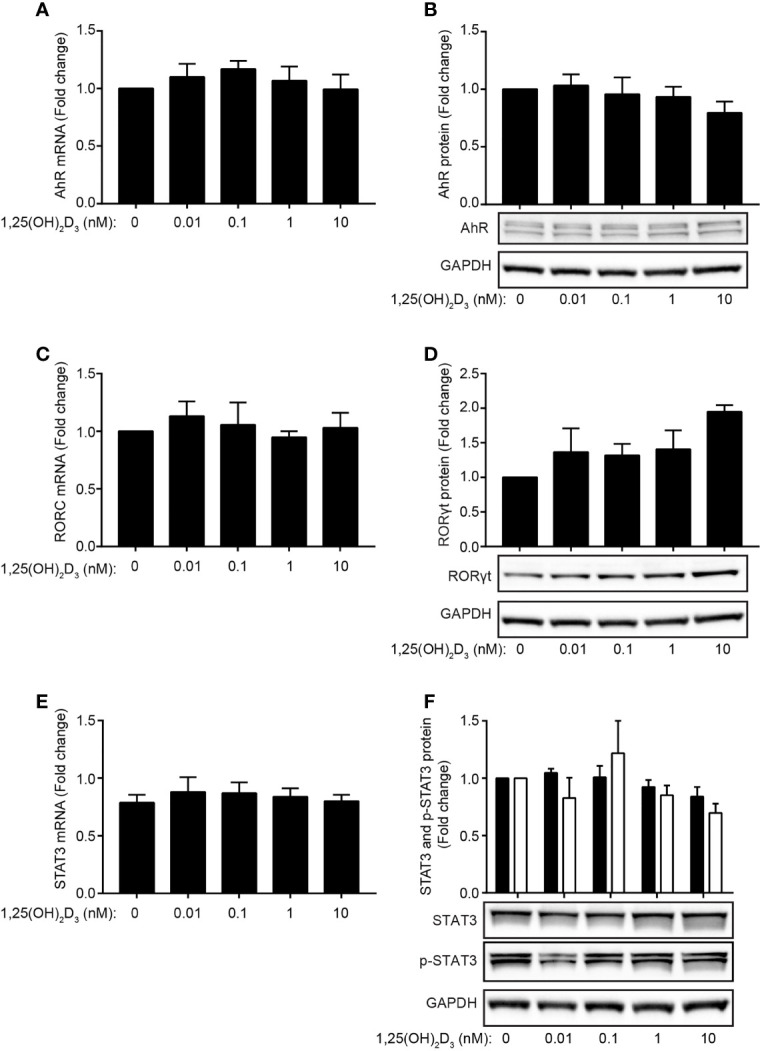
1,25(OH)_2_D_3_ does not inhibit IL-22 by affecting AhR, RORγt or STAT-3 expression. **(A)** Relative AhR mRNA expression and **(B)** representative Western Blot (lower panel) and quantification (upper panel) of AhR with GAPDH as loading control from naïve CD4^+^ T cells co-cultured with allogeneic DC for 96 h in Th22 medium and the indicated concentrations of 1,25(OH)_2_D_3_. **(C)** Relative RORC mRNA expression and **(D)** representative Western Blot (lower panel) and quantification (upper panel) of RORγt with GAPDH as loading control from naïve CD4^+^ T cells co-cultured with allogeneic DC for 96 h in Th22 medium and the indicated concentrations of 1,25(OH)_2_D_3_. **(E)** Relative STAT-3 mRNA expression and **(F)** representative Western Blot (lower panel) and quantification (upper panel) of STAT-3 and phosphorylated STAT-3 (p-STAT3) with GAPDH as loading control from naïve CD4^+^ T cells co-cultured with allogeneic DC for 96 h in Th22 medium and the indicated concentrations of 1,25(OH)_2_D_3_. The data are normalized to the values obtained from DC-T cell co-cultures incubated in Th22 medium in the absence of 1,25(OH)_2_D_3_. (mean + SEM, two experiment with 4 donors).

Recently, it has been reported that the transcription factors AhR, RORγt and STAT-3 play critical roles in IL-21-mediated induction of IL-22 in mouse T cells (29). In the present study, we found that the transcription factors AhR and RORγt regulate IL-22 secretion in human Th22 cells. To investigate whether 1,25(OH)_2_D_3_ indirectly inhibits IL-22 production in human Th22 cells through down-regulation of AhR, RORγt or STAT-3, we activated naïve CD4^+^ T cells with allogeneic DC in Th22 medium in the absence or presence of 1,25(OH)_2_D_3_. After 96 h of culture, we determined the mRNA and protein expression levels of AhR, RORγt and STAT-3. We found that the mRNA and protein expression levels of AhR (**Figures 4A, B**), RORγt (**Figures 4C, D**) and STAT-3 (**Figures 4E, F**) were not significantly affected by 1,25(OH)_2_D_3_. Furthermore, 1,25(OH)_2_D_3_ did not significantly affect the phosphorylation of STAT-3 (**Figure 4F**). Thus, 1,25(OH)_2_D_3_ did not inhibit IL-22 expression and production by inhibition of AhR, RORγt or STAT-3 expression or STAT-3 phosphorylation in human Th22 cells.

### IL-21 Does Not Rescue IL-22 Production in 1,25(OH)_2_D_3_-Treated Th22 Cells

**Figure 5:**
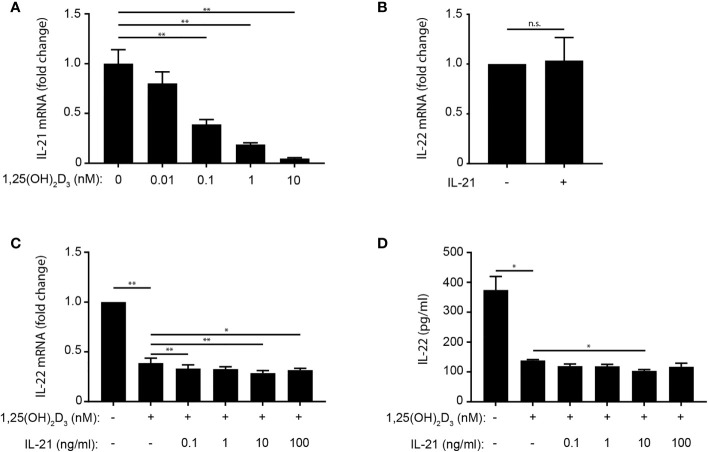
IL-21 does not rescue IL-22 production in 1,25(OH)_2_D_3_-treated Th22 cells. **(A)** Relative IL-21 mRNA expression in naïve CD4^+^ T cells co-cultured with allogeneic DC for 96 h in Th22 medium and the indicated concentrations of 1,25(OH)_2_D_3_. Data are normalized to the values obtained from to DC-T cell co-cultures incubated in Th22 medium in the absence of 1,25(OH)_2_D_3_. **(B)** IL-22 in the supernatant of naïve CD4^+^ T cells activated with allogeneic DC for 96 h in Th22 medium in the absence or presence of IL-21 (10 ng/ml). Data are normalized to the values obtained from to DC-T cell co-cultures incubated in Th22 medium in the absence of IL-21. **(C)** Relative IL-22 mRNA expression and **(D)** IL-22 production in naïve CD4^+^ T cells activated with allogeneic DC for 96 h in Th22 medium in the absence or presence of 1,25(OH)_2_D_3_ (10 nM) and the indicated concentrations of IL-21. The data in **(C)** are normalized to the values obtained from to DC-T cell co-cultures incubated in Th22 medium in the absence of 1,25(OH)_2_D_3_ and IL-21. **(A–D)** Mean + SEM from one experiment with 4 donors. n.s., not significant.

In mice, IL-21 promotes IL-22 production in CD4^+^ T cells (29). To investigated whether 1,25(OH)_2_D_3_ regulate IL-21 in human Th22 cells, we activated naïve CD4^+^ T cells in Th22 medium in the absence or presence of 1,25(OH)_2_D_3_ and measured IL-21 concentration in the supernatant at day 4. We found that 1,25(OH)_2_D_3_ down-regulated IL-21 mRNA and protein expression in human Th22 cells (**Figure 5A** and **Supplementary Figure 4A**). Even though we did not find IL-21 to up-regulate IL-22 in the absence of 1,25(OH)2D3 (**Figure 5B**), the possibility existed that 1,25(OH)_2_D_3_ indirectly inhibited IL-22 production by inhibition of IL-21. If this was the case exogenous IL-21 should rescue 1,25(OH)_2_D_3_-mediated IL-22 inhibition. Consequently, we activated naïve CD4^+^ T cells with allogeneic DC in Th22 medium in the absence or presence of 1,25(OH)_2_D_3_ and increasing concentrations of exogenous IL-21. After 96 h of culture, we determined IL-22 mRNA expression and IL-22 secretion. We found that IL-21 did neither rescue IL-22 mRNA expression nor IL-22 secretion in Th22 cells treated with 1,25(OH)_2_D_3_ (**Figures 5C, D**). Likewise, we found that anti-IL-21 antibodies did not inhibit IL-22 production although it strongly neutralised IL-21 in the culture supernatants (**Supplementary Figures 4A, B**).

### 1,25(OH)_2_D_3_ Inhibits IL-22 Production in the CTCL Cell Line Myla 2059

**Figure 6:**
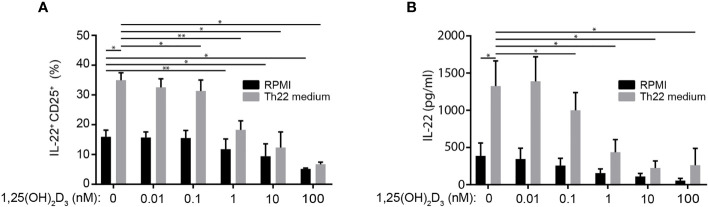
1,25(OH)_2_D_3_ inhibits IL-22 production in the CTCL cell line Myla 2059. **(A)** Frequency of IL-22^+^ Myla 2059 cell and **(B)** IL-22 in the supernatant of Myla 2059 cells incubation for 48 h in the absence (black columns) or presence of Th22 medium (white columns) and in the presence of the indicated concentrations of 1,25(OH)_2_D_3_ (mean + SEM, two independent experiments with 4 donors).

IL-22 is highly expressed and involved in the establishment of the pro-tumorigenic environment in the skin of patients with CTCL (47). To investigate the effect of Th22 medium and 1,25(OH)_2_D_3_ on IL-22 expression in CTCL cells, we cultured the CTCL cell line Myla 2059 in the absence or presence of 1,25(OH)_2_D_3_ in RPMI in the absence or presence of the Th22 promoting factors as defined in the Th22 medium. After 48 h of culture, we measured the frequency of IL-22^+^ Myla 2059 cells and the IL-22 concentration in the supernatants. We found that the Th22 promoting factors significantly increased the proportion of IL-22^+^ Myla 2059 cells and IL-22 production (**Figures 6A, B**, for gating strategy please see **Supplementary Figure 1B**). Furthermore, we found that 1,25(OH)_2_D_3_ inhibited the frequency of IL-22^+^ Myla 2059 cells (**Figures 6A** and **S1B**). Likewise, 1,25(OH)_2_D_3_ inhibited the production of IL-22 from Myla 2059 cells both in the absence and presence of Th22 promoting factors (**Figure 6B**). Taken together, these data showed that Th22 promoting factors increased IL-22 production in Myla 2059 cells and that 1,25(OH)_2_D_3_ inhibited IL-22 production in Myla 2059 cells as seen in Th22 cells.

### 1,25(OH)_2_D_3_ Inhibits IL-22 Production *via* a Repressive VDRE in the *il22* Promoter

**Figure 7:**
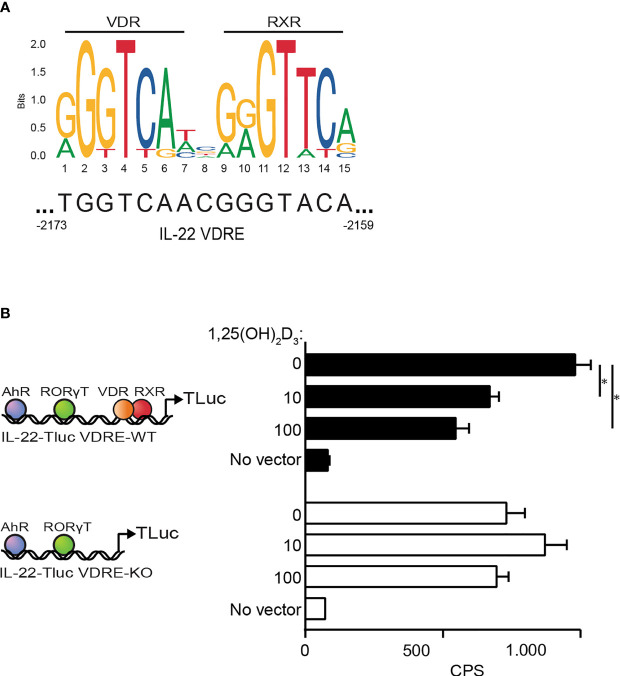
1,25(OH)_2_D_3_ inhibits IL-22 production *via a* repressive VDRE in the *il22* promoter **(A)** Schematic representation of the transcription factor VDR : RXR and the DNA binding consensus motif as position frequency matrices for VDR : RXR in homo sapiens found in the open-access database JASPAR (48, 49). **(B)** (Left) Schematic representation of the plasmid construct IL-22-Tluc with the VDRE (IL-22-TLuc VDRE-WT) located at 2159-2173 base pairs upstream the start site of the *il22* gene and the plasmid construct IL-22-Tluc with the VDRE deletion (IL-22-TLuc VDRE-KO). (Right) Luciferase light emission in counts per second (CPS) in Myla 2059 cells nucleofected either with IL-22-TLuc VDRE-WT (black) or IL-22-TLuc VDRE-KO (white) plasmids and cultured for 48 h in the presence of 1,25(OH)_2_D_3_ at the indicated concentrations in nM (mean + SEM, three independent experiments).

The observations described above suggested that the 1,25(OH)_2_D_3_-induced repression of IL-22 transcription was not indirectly mediated by inhibition of transcription factors but was a direct effect of 1,25(OH)_2_D_3_ on the *il22* gene. Consequently, we searched for potential VDRE in the *il22* promoter using the JASPAR database of transcription factor binding profiles (48). We found a potential VDRE sequence in the *il22* promoter located 2159-2173 base pairs upstream from the start codon of the *il22* gene (**Figure 7A**). To determine whether this sequence actually represented a repressive VDRE, we constructed two reporter vectors where luciferase expression was dependent on the *il22* promoter. One of the vectors contained the wild-type *il22* promoter sequence including the putative VDRE (IL22-TLuc VDRE-WT) and the other vector contained the *il22* promoter sequence where the putative VDRE was deleted (IL22-TLuc VDRE-KO) (**Figure 7B**). We transfected Myla 2059 cells with the vectors and compared luciferase light emission in untreated cells and in cells treated with 1,25(OH)_2_D_3_. We found that 1,25(OH)_2_D_3_ inhibited luciferase light emission in Myla 2059 cells transfected with the IL22-TLuc VDRE-WT vector but not in Myla 2059 cells transfected with the IL22-TLuc VDRE-KO vector (**Figure 7B**). These data indicated that the *il22* promoter contains a repressive VDRE located 2159-2173 base pairs upstream from the start codon of the *il22* gene.

## Discussion

In this study, we show that 1,25(OH)_2_D_3_ inhibits IL-22 expression and production in human Th22 cells through a repressive VDRE in the *il22* promoter. 1,25(OH)_2_D_3_ is well-known by its immunomodulatory properties and it can influence the differentiation of T helper cells by regulating the production of their signature cytokine (35–41). Some studies have investigated the effect of 1,25(OH)_2_D_3_ on IL-22 in human and mice CD4^+^ T cells (4, 42, 43). However, conflicting results were obtained. One study found that 1,25(OH)_2_D_3_ inhibited IL-22 production in human Th17 cells (43), whereas others found that 1,25(OH)_2_D_3_ promoted Th22 cell differentiation and IL-22 production (4, 42). Thus, the effect of 1,25(OH)_2_D_3_ on IL-22 in human Th22 cells remained to be fully elucidated. In the present study we demonstrate that 1,25(OH)_2_D_3_ strongly inhibited IL-22 production in Th22 cells. This was not caused by 1,25(OH)_2_D_3_-mediated inhibition of the expression of the transcription factors AhR, RORγt and STAT-3 or by the inhibition of IL-21 production. Although we did not formally rule out that 1,25(OH)_2_D_3_ might affect the binding of those transcription factors to target genes, we show that 1,25(OH)_2_D_3_ directly inhibits IL-22 production through a repressive VDRE located in the *il22* promoter. This is in line with previous studies that identified repressive VDRE in the *ifng* (50) and *il12B* (51) promoters.

In the present study, we describe a novel way to differentiate human Th22 cells *in vitro.* In our system, where we activate naïve CD4^+^ T cells with allogeneic dendritic cells, the sole presence of IL-6 and TNFα did not increase IL-22 production. However, we found that IL-6, TNFα, IL-1β and IL-23 lead to an increase in both IL-22 and IL-17 production. This is in accordance to previous studies that found that these factors increase IL-17 production in CD4^+^ T cells (52–54). As the AhR agonist FICZ has been found to induce IL-22 and inhibit IL-17, we included FICZ in the Th22 panel. We observed that FICZ lead to increased IL-22 production while down-regulating IL-17. However, IL-17 was still produced to some extent in the presence of FICZ. A key cytokine in the differentiation of Th17 cells is TGFβ (55–57). Thus, we included an inhibitor of TGFβR signalling (galunisertib) in an attempt to repress the generation of IL-17-producing CD4^+^ T cells in the presence of factors that induce IL-22 production. Interestingly, we found that galunisertib augmented the production of IL-22 while inhibiting IL-17 production. Taken together, we found that the combination of IL-6, TNFα, IL-1β, IL-23, FICZ and galunisertib constituted optimal conditions for *in vitro* generation of human Th22 cells.

We found that AhR and RORγt are important transcription factors that regulate IL-22 in human Th22 cells. In accordance, AhR and RORγt regulate IL-22 expression and production in ILC3 that represent a major IL-22 source in the gut (58, 59). In contrast to a recent study that identified IL-21 as an inducer of IL-22 production in mouse CD4^+^ T cells (29), we found that IL-21 do not affect IL-22 production in human Th22 cells. Thus, our data suggest that regulation of IL-22 may differ between mice and human CD4^+^ T cells.

Interestingly, ectopic IL-22 expression is a characteristic feature of lesional skin in CTCL, and IL-22 is believed to play a role in the establishment of the pro-tumorigenic microenvironment and the deficient antimicrobial defence in these patients (47, 60). Of notice, CTCL lesions are often localized to body areas, which are not exposed to sunlight i.e. the “bathing suit area” and in general, CTCL patients display deficient vitamin D serum levels (49). Given the present findings that vitamin D inhibit IL-22 expression in malignant T cells, we hypothesize that vitamin D supplementation could have a beneficial effect as adjuvant therapy inhibiting ectopic IL-22 expression and skin inflammation in CTCL.

In conclusion, we have identified a novel way of differentiating naïve CD4^+^ T cells towards the Th22 lineage and demonstrated that 1,25(OH)_2_D_3_ directly inhibits IL-22 through VDR targeting a repressive VDRE located in the *il22* gene. We showed that AhR and RORγt regulate IL-22 in human Th22 cells, whereas IL-21 does not affect IL-22 production in human Th22 cells. This study add to the understanding on IL-22 regulation in human Th22 cells and suggests that vitamin D may be considered a potential therapeutics to regulate IL-22-mediated diseases.

## Data Availability Statement

The raw data supporting the conclusions of this article will be made available by the authors, without undue reservation.

## Ethics Statement

The studies involving human participants were reviewed and approved by Regional Ethical Committee of the Capital Region of Denmark. The patients/participants provided their written informed consent to participate in this study.

## Author Contributions

CG, MK-W and DL conceived the study and designed the experiments. DL, FA-J, ND, UP and ST performed the laboratory experiments. CB, BW, AW and NØ assisted with the experimental design and data interpretation. CG, MK-W and DL analysed the data and wrote the manuscript with input from all authors. All authors contributed to the article and approved the submitted version.

## Funding

This work was supported by the LEO Foundation (LF17058).

## Conflict of Interest

The authors declare that the research was conducted in the absence of any commercial or financial relationships that could be construed as a potential conflict of interest.

## Publisher’s Note

All claims expressed in this article are solely those of the authors and do not necessarily represent those of their affiliated organizations, or those of the publisher, the editors and the reviewers. Any product that may be evaluated in this article, or claim that may be made by its manufacturer, is not guaranteed or endorsed by the publisher.

## References

[1] Zhu J Yamane H Paul WE. Differentiation of Effector CD4 T Cell Populations. Annu Rev Immunol (2010) 28:445–89. 10.1146/annurev-immunol-030409-101212 PMC3502616 20192806

[2] Zhu J. T Helper Cell Differentiation, Heterogeneity, and Plasticity. Cold Spring Harb Perspect Biol (2018) 10:a030338. 10.1101/cshperspect.a030338 28847903 PMC6169815

[3] Eyerich S Eyerich K Pennino D Carbone T Nasorri F Pallotta S. Th22 Cells Represent a Distinct Human T Cell Subset Involved in Epidermal Immunity and Remodeling. J Clin Invest (2009) 119:3573–85. 10.1172/JCI40202 PMC2786807 19920355

[4] Duhen T Geiger R Jarrossay D Lanzavecchia A Sallusto F. Production of Interleukin 22 But Not Interleukin 17 by a Subset of Human Skin-Homing Memory T Cells. Nat Immunol (2009) 10:857–63. 10.1038/ni.1767 19578369

[5] Wolk K Witte E Witte K Warszawska K Sabat R. Biology of Interleukin-22. Semin Immunopathol (2010) 32:17–31. 10.1007/s00281-009-0188-x 20127093

[6] Wolk K Sabat R. Interleukin-22: A Novel T- and NK-Cell Derived Cytokine That Regulates the Biology of Tissue Cells. Cytokine Growth Factor Rev (2006) 17:367–80. 10.1016/j.cytogfr.2006.09.001 17030002

[7] Wolk K Kunz S Witte E Friedrich M Asadullah K Sabat R. IL-22 Increases the Innate Immunity of Tissues. Immunity (2004) 21:241–54. 10.1016/j.immuni.2004.07.007 15308104

[8] Wolk K Witte E Wallace E Döcke WD Kunz S Asadullah K. IL-22 Regulates the Expression of Genes Responsible for Antimicrobial Defense, Celular Differentiation and Mobility in Keratinocytes: A Potential Role in Psoriasis. Eur J Immunol (2006) 36:1309. 10.1002/eji.200535503 16619290

[9] Pham TA Clare S Goulding D Arasteh JM Stares MD Browne HP. Epithelial IL-22RA1-Mediated Fucosylation Promotes Intestinal Colonization Resistance to an Opportunistic Pathogen. Cell Host Microbe (2014) 16:504–16. 10.1016/j.chom.2014.08.017 PMC4190086 25263220

[10] Liang SC Tan XY Luxenberg DP Karim R Dunussi-Joannopoulos K Collins M. Interleukin (IL)-22 and IL-17 Are Coexpressed by Th17 Cells and Cooperatively Enhance Expression of Antimicrobial Peptides. J Exp Med (2006) 203:2271–9. 10.1084/jem.20061308 PMC2118116 16982811

[11] Lindemans CA Calafiore M Mertelsmann AM O'Connor MH Dudakov JA Jenq RR. Interleukin-22 Promotes Intestinal Stem Cell-Mediated Epithelial Re-Generation. Nature (2016) 528:560–4. 10.1038/nature16460 PMC4720437 26649819

[12] Boniface K Bernard FX Garcia M Gurney AL Lecron JC Morel F. IL-22 Inhibits Epidermal Differentiation and Induces Proinflammatory Gene Expression and Migration of Human Keratinocytes. J Immunol (2005) 174:3695–702. 10.4049/jimmunol.174.6.3695 15749908

[13] Nograles KE Zaba LC Guttman-Yassky E Fuentes-Duculan J Suárez-Fariñas M Cardinale I. Th17 Cytokines Interleukin (IL)-17 and IL-22 Modulate Distinct Inflammatory and Keratinocyte-Response Pathways. Br J Dermatol (2008) 159(5):1092–102. 10.1111/j.1365-2133.2008.08769.x PMC2724264 18684158

[14] Ahn K Kim BE Kim J Leung DY. Recent Advances in Atopic Dermatitis. Curr Opin Immunol (2020) 66:14–21. 10.1016/j.coi.2020.02.007 32299014 PMC7554175

[15] Jin M Yoon J. From Bench to Clinic: The Potential of Therapeutic Targeting of the IL-22 Signaling Pathway in Atopic Dermatitis. Immune Netw (2018) 18:e42. 10.4110/in.2018.18.e42 30619628 PMC6312894

[16] Eberle FC Bruck J Holstein J Hirahara K Ghoreschi K. Recent Advances in Understanding Psoriasis. F1000Res (2016) 5:F1000. 10.12688/f1000research.7927.1 PMC4850872 27158469

[17] Cui G. TH9, TH17, and TH22 Cell Subsets and Their Main Cytokine Products in the Pathogenesis of Colorectal Cancer. Front Oncol (2019) 9:1002. 10.3389/fonc.2019.01002 31637216 PMC6787935

[18] Doulabi H Rastin M Shabahangh H Maddah G Abdollahi A Nosratabadi R. Analysis of Th22, Th17 and CD4+cells Co-Producing IL-17/IL-22 at Different Stages of Human Colon Cancer. BioMed Pharmacother (2018) 103:1101–6. 10.1016/j.biopha.2018.04.147 29710675

[19] Shen E Wang M Xie H Zou R Lin Q Lai L. Existence of Th22 in Children and Evaluation of IL-22 + CD4 + T, Th17, and Other T Cell Effector Subsets From Healthy Children Compared to Adults. BMC Immunol (2016) 17:20. 10.1186/s12865-016-0158-8 27338754 PMC4918114

[20] Trifari S Kaplan CD Tran EH Crellin NK Spits H. Identification of a Human Helper T Cell Population That Has Abundant Production of Interleukin 22 and Is Distinct From TH-17, TH1 and TH2 Cells. Nat Immunol (2009) 10:864–71. 10.1038/ni.1770 19578368

[21] Plank MW Kaiko GE Maltby S Weaver J Tay HL Shen W. Th22 Cells Form a Distinct Th Lineage From Th17 Cells In Vitro With Unique Transcriptional Properties and Tbet-Dependent Th1 Plasticity. J Immunol (2017) 198:2182–90. 10.4049/jimmunol.1601480 PMC5367520 28100680

[22] Kara EE Comerford I Fenix KA Bastow CR Gregor CE McKenzie DR. Tailored Immune Responses: Novel Effector Helper T Cell Subsets in Protective Immunity. PloS Pathog (2014) 10:e1003905. 10.1371/journal.ppat.1003905 24586147 PMC3930558

[23] Wolk K Kunz S Asadullah K Sabat R. Cutting Edge: Immune Cells as Sources and Targets of the IL-10 Family Members? J Immunol (2002) 168:5397–402. 10.4049/jimmunol.168.11.5397 12023331

[24] Witte E Witte K Warszawska K Sabat R Wolk K. Interleukin-22: A Cytokine Produced by T, NK and NKT Cell Subsets, With Importance in the Innate Immune Defense and Tissue Protection. Cytokine Growth Factor Rev (2010) 21:365–79. 10.1016/j.cytogfr.2010.08.002 20870448

[25] Qiu J Heller JJ Guo X Chen ZM Fish K Fu YX. The Aryl Hydrocarbon Receptor Regulates Gut Immunity Through Modulation of Innate Lymphoid Cells. Immunity (2012) 36:92–104. 10.1016/j.immuni.2011.11.011 22177117 PMC3268875

[26] Cording S Medvedovic J Cherrier M Eberl G. Development and Regulation of RORγt+ Innate Lymphoid Cells. FEBS Lett (2014) 588:4176–81. 10.1016/j.febslet.2014.03.034 24681095

[27] Hazenberg MD Spits H. Human Innate Lymphoid Cells. Blood (2014) 124:700–9. 10.1182/blood-2013-11-427781 24778151

[28] Sanos SL Bui VL Mortha A Oberle K Heners C Johner C. RORgammat and Commensal Microflora Are Required for the Differentiation of Mucosal Interleukin 22-Producing NKp46+ Cells. Nat Immunol (2009) 10:83–91. 10.1038/ni.1684 19029903 PMC4217274

[29] Yeste A Mascanfroni ID Nadeau M Burns EJ Tukpah AM Santiago A. IL-21 Induces IL-22 Production in CD4+ T Cells. Nat Commun (2014) 5:3753. 10.1038/ncomms4753 24796415 PMC4157605

[30] Pike JW Meyer MB Bishop KA. Regulation of Target Gene Expression by the Vitamin D Receptor - an Update on Mechanisms. Rev Endocr Metab Disord (2012) 13:45–55. 10.1007/s11154-011-9198-9 21870057

[31] Haussler MR Whitfield GK Kaneko I Haussler CA Hsieh D Hsieh JC. Molecular Mechanisms of Vitamin D Action. Calcif Tissue Int (2013) 92:77–98. 10.1007/s00223-012-9619-0 22782502

[32] Haussler MR Whitfield GK Haussler CA Hsieh JC Thompson PD Selznick SH. The Nuclear Vitamin D Receptor: Biological and Molecular Regulatory Properties Revealed. In: Journal of Bone and Mineral Research. The Official Journal of the American Society for Bone and Mineral Research (1998) 13(3):325–49. 10.1359/jbmr.1998.13.3.325 9525333

[33] Jurutka PW Hsieh J-C Remus LS Whitfield GK Thompson PD Haussler CA. Mutations in the 1,25-Dihydroxyvitamin D3 Receptor Identifying C-Terminal Amino Acids Required for Transcriptional Activation That Are Functionally Dissociated From Hormone Binding, Heterodimeric DNA Binding and Interaction With Basal Transcription Factor IIB, In Vitro. J Biol Chem (1997) 272:14592–9. 10.1074/jbc.272.23.14592 9169418

[34] Thompson PD Remus LS Hsieh J-C Jurutka PW Whitfield GK Galligan MA. Distinct Retinoid X Receptor Activation Function-2 Residues Mediate Transactivation in Homodimeric and Vitamin D Receptor Heterodimeric Contexts. J Mol Endocrinol (2001) 27:211–27. 10.1677/jme.0.0270211 11564604

[35] Adams JS Rafison B Witzel S Reyes RE Shieh A Chun R. Regulation of the Extrarenal CYP27B1-Hydroxylase. J Steroid Biochem Mol Biol (2014) 144:22–7. 10.1016/j.jsbmb.2013.12.009 PMC4077994 24388948

[36] Nagpal S Na S Rathnachalam R. Noncalcemic Actions of Vitamin D Receptor Ligands. Endocr Rev (2005) 26:662–87. 10.1210/er.2004-0002 15798098

[37] Kongsbak M von Essen MR Boding L Levring TB Schjerling P Lauritsen JP. Vitamin D Up-Regulates the Vitamin D Receptor by Protecting it From Proteasomal Degradation in Human CD4+ T Cells. PloS One (2014) 9:e96695. 10.1371/journal.pone.0096695 24792400 PMC4008591

[38] Rigby WF Yirinec B Oldershaw RL Fanger MW. Comparison of the Effects of 1,25-Dihydroxyvitamin D3 on T Lymphocyte Subpopulations. Eur J Immunol (1987) 17:563–6. 10.1002/eji.1830170420 3106071

[39] Jeffery LE Burke F Mura M Zheng Y Qureshi OS Hewison M. 1,25-Dihydroxyvitamin D3 and IL-2 Combine to Inhibit T Cell Production of Inflammatory Cytokines and Promote Development of Regulatory T Cells Expressing CTLA-4 and FoxP3. J Immunol (2009) 183:5458–67. 10.4049/jimmunol.0803217 PMC2810518 19843932

[40] van Etten E Mathieu C. Immunoregulation by 1,25-Dihydroxyvitamin D3: Basic Concepts. J Steroid Biochem Mol Biol (2005) 97:93–101. 10.1016/j.jsbmb.2005.06.002 16046118

[41] Palmer MT Lee YK Maynard CL Oliver JR Bikle DD Jetten AM. Lineage-Specific Effects of 1,25-Dihydroxyvitamin D(3) on the Development of Effector CD4 T Cells. J Biol Chem (2011) 286:997–1004. 10.1074/jbc.M110.163790 21047796 PMC3020784

[42] Sommer A Fabri M. Vitamin D Regulates Cytokine Patterns Secreted by Dendritic Cells to Promote Differentiation of IL-22-Producing T Cells. PloS One (2015) 10:e0130395. 10.1371/journal.pone.0130395 26107738 PMC4480856

[43] Lovato P Norsgaard H Tokura Y Røpke MA. Calcipotriol and Betamethasone Dipropionate Exert Additive Inhibitory Effects on the Cytokine Expression of Inflammatory Dendritic Cell-Th17 Cell Axis in Psoriasis. J Dermatol Sci (2017) 85:147. 10.1016/j.jdermsci.2015.12.009 27964877

[44] Woetmann A Lovato P Eriksen KW Krejsgaard T Labuda T Zhang Q. Nonmalignant T Cells Stimulate Growth of T-Cell Lymphoma Cells in the Presence of Bacterial Toxins. Blood (2007) 109:3325–32. 10.1182/blood-2006-04-017863 PMC1852254 17179233

[45] Available at: http://jaspar.genereg.net/matrix/MA0074.1/.

[46] Al-Jaberi FAH Kongsbak-Wismann M Aguayo-Orozco A Krogh N Buss T Lopez DV. Impaired Vitamin D Signaling in T Cells From a Family With Hereditary Vitamin D Resistant Rickets. Front Immunol (2021) 12:684015. 10.3389/fimmu.2021.684015 34093587 PMC8170129

[47] Miyagaki T Sugaya M Suga H Kamata M Ohmatsu H Fujita H. IL-22, But Not IL-17, Dominant Environment in Cutaneous T-Cell Lymphoma. Clin Cancer Res (2011) 17:7529–38. 10.1158/1078-0432.CCR-11-1192 22048239

[48] Fornes O Castro-Mondragon JA Khan A van der Lee R Zhang X Richmond PA. JASPAR 2020: Update of the Open-Access Database of Transcription Factor Binding Profiles. Nucleic Acids Res (2020) 48:D87–92. 10.1093/nar/gkz1001 PMC7145627 31701148

[49] Talpur R Cox KM Hu M Geddes ER Parker MK Yang BY. Vitamin D Deficiency in Mycosis Fungoides and Sézary Syndrome Patients Is Similar to Other Cancer Patients. Clin Lymphoma Myeloma Leuk (2014) 14(6):518–24. 10.1016/j.clml.2014.06.023 25442486

[50] Cippitelli M Santoni A. Vitamin D3: A Transcriptional Modulator of the Interferon-Gamma Gene. Eur J Immunol (1998) 28:3017–30. 10.1002/(SICI)1521-4141(199810)28:10<3017::AID-IMMU3017>3.0.CO;2-6 9808170

[51] Gynther P Toropainen S Matilainen JH Seuter S Carlberg C Väisänen S. Mechanism of 1α,25-Dihydroxyvitamin D3-Dependent Repression of Interleu-Kin-12B. Biochim Biophys Acta (2011) 1813:810–8. 10.1016/j.bbamcr.2011.01.037 21310195

[52] Chung Y Chang SH Martinez GJ Yang XO Nurieva R Kang HS. Critical Regulation of Early Th17 Cell Differentiation by Interleukin-1 Signaling. Immunity (2009) 30:576–87. 10.1016/j.immuni.2009.02.007 PMC2705871 19362022

[53] Sutton C Brereton C Keogh B Mills KH Lavelle EC. A Crucial Role for Interleukin (IL)-1 in the Induction of IL-17-Producing T Cells That Mediate Autoimmune Encephalomyelitis. J Exp Med (2006) 203:1685–91. 10.1084/jem.20060285 PMC2118338 16818675

[54] Ivanov II McKenzie BS Zhou L Tadokoro CE Lepelley A Lafaille JJ. The Orphan Nuclear Receptor RORgammat Directs the Differentiation Program of Proinflammatory IL-17+ T Helper Cells. Cell (2006) 126:1121–33. 10.1016/j.cell.2006.07.035 16990136

[55] Mangan PR Harrington LE O’Quinn DB Helms WS Bullard DC Elson CO. Transforming Growth Factor-Beta Induces Development of the T(H)17 Lineage. Nature (2006) 441:231–4. 10.1038/nature04754 16648837

[56] Veldhoen M Hocking RJ Atkins CJ Locksley RM Stockinger B. TGFbeta in the Context of an Inflammatory Cytokine Milieu Supports *De Novo* Differentiation of IL-17-Producing T Cells. Immunity (2006) 24:179–89. 10.1016/j.immuni.2006.01.001 16473830

[57] Morishima N Mizoguchi I Takeda K Mizuguchi J Yoshimoto T. TGF-Beta Is Necessary for Induction of IL-23R and Th17 Differentiation by IL-6 and IL-23. Biochem Biophys Res Commun (2009) 386:105–10. 10.1016/j.bbrc.2009.05.140 19501566

[58] Lee JS Cella M McDonald KG Garlanda C Kennedy GD Nukaya M. AHR Drives the Development of Gut ILC22 Cells and Postnatal Lymphoid Tissues *Via* Pathways Dependent on and Independent of Notch. Nat Immunol (2011) 13:144–51. 10.1038/ni.2187 PMC3468413 22101730

[59] Tripathi D Radhakrishnan RK Sivangala Thandi R Paidipally P Devalraju KP Neela VSK. IL-22 Produced by Type 3 Innate Lymphoid Cells (ILC3s) Reduces the Mortality of Type 2 Diabetes Mellitus (T2DM) Mice Infected With Mycobacterium Tuberculosis. PloS Pathog (2019) 15:e1008140. 10.1371/journal.ppat.1008140 31809521 PMC6919622

[60] Papathemeli D Patsatsi A Papanastassiou D Koletsa T Papathemelis T Avgeros C. Protein and mRNA Expression Levels of Interleukin-17A, -17F and -22 in Blood and Skin Samples of Patients With Mycosis Fungoides. Acta Derm Venereol (2020) 100(18):adv00326. 10.2340/00015555-3688 33170303 PMC9309846

