# Supplementary material for: Vitamin D Inhibits IL-22 Production Through a Repressive Vitamin D Response Element in the il22 Promoter
Source: Front Immunol. 2021 Aug 2;12:715059. doi: 10.3389/fimmu.2021.715059 (PMC8366496; doi:10.3389/fimmu.2021.715059)
Supplement: Supplementary file 1 [file Image_1.pdf]

Supplementary Figure 1
Villalba Lopez et al.

A

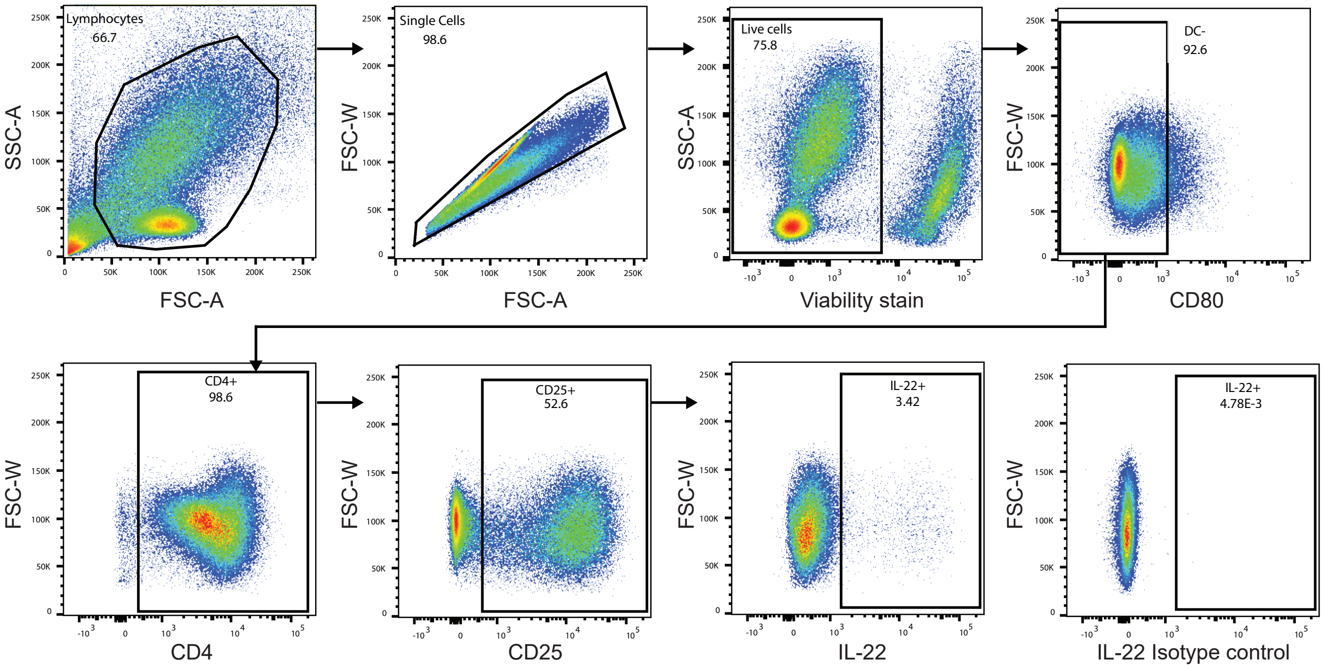

B

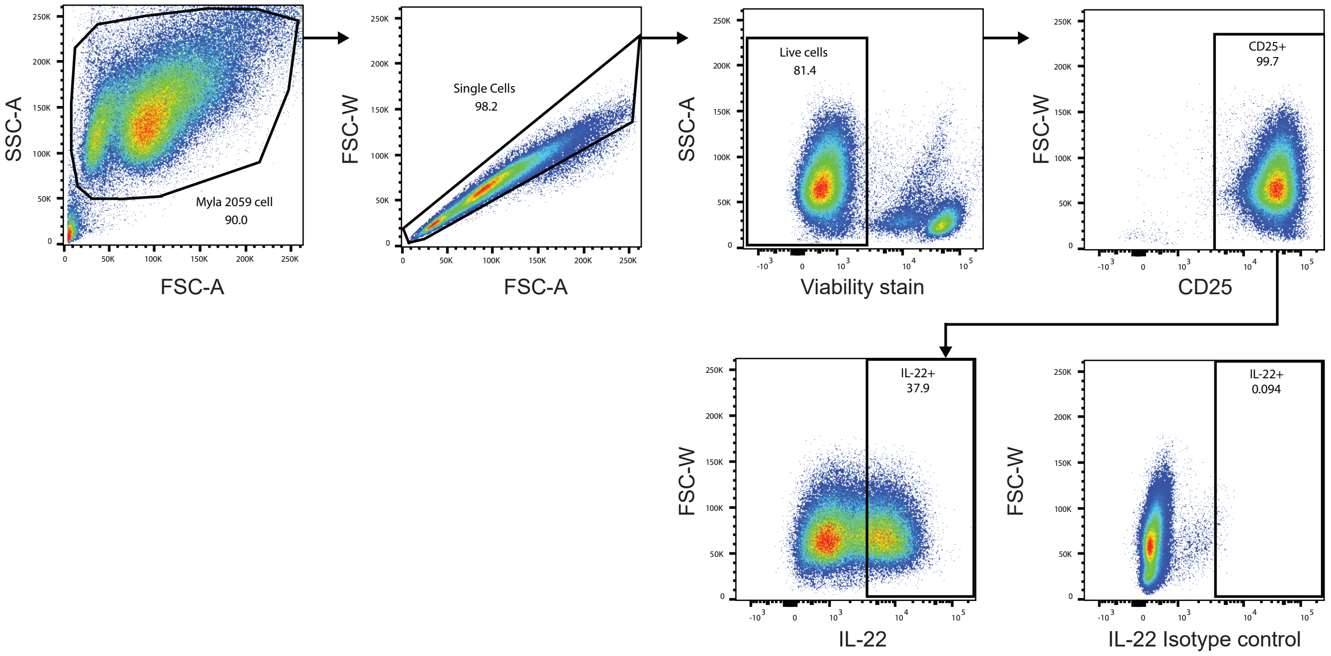

SUPPLEMENTARY FIGURE 1. Gating strategy used to determine the frequency of **(A)** IL-22^{+} CD25^{+} Th22 cells and **(B)** IL-22^{+} CD25^{+} Myla 2059 cells.
