# Supplementary material for: Vitamin D Inhibits IL-22 Production Through a Repressive Vitamin D Response Element in the il22 Promoter
Source: Front Immunol. 2021 Aug 2;12:715059. doi: 10.3389/fimmu.2021.715059 (PMC8366496; doi:10.3389/fimmu.2021.715059)
Supplement: Supplementary file 2 [file Image_2.pdf]

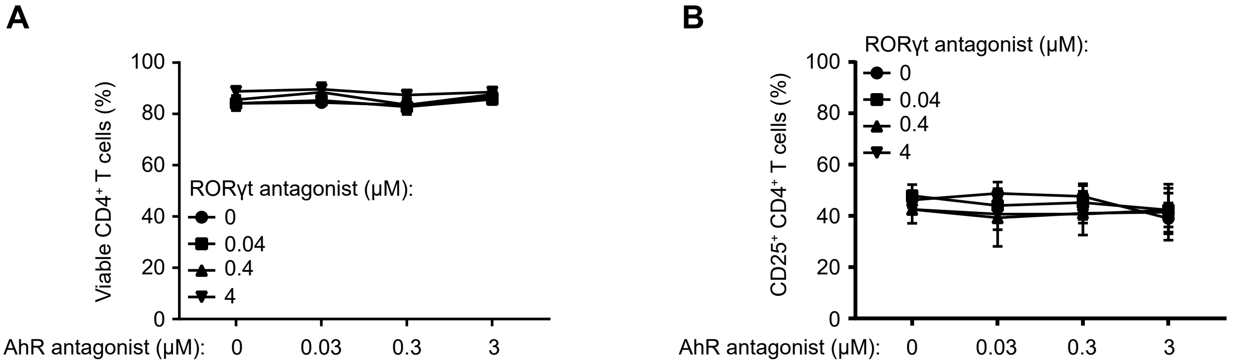

SUPPLEMENTARY FIGURE 2. (A) Cell viability and **(B)** expression of the activation marker CD25 on $CD4^+$ T cells stimulated in Th22 medium for 96 h with the indicated concentrations of the AhR antagonist CH-223191 and the RORγt antagonist SR-2211. Data were obtained from one experiment with two donors.
