# Supplementary material for: Vitamin D Inhibits IL-22 Production Through a Repressive Vitamin D Response Element in the il22 Promoter
Source: Front Immunol. 2021 Aug 2;12:715059. doi: 10.3389/fimmu.2021.715059 (PMC8366496; doi:10.3389/fimmu.2021.715059)
Supplement: Supplementary file 3 [file Image_3.pdf]

Supplementary Figure 3
Villalba Lopez et al.

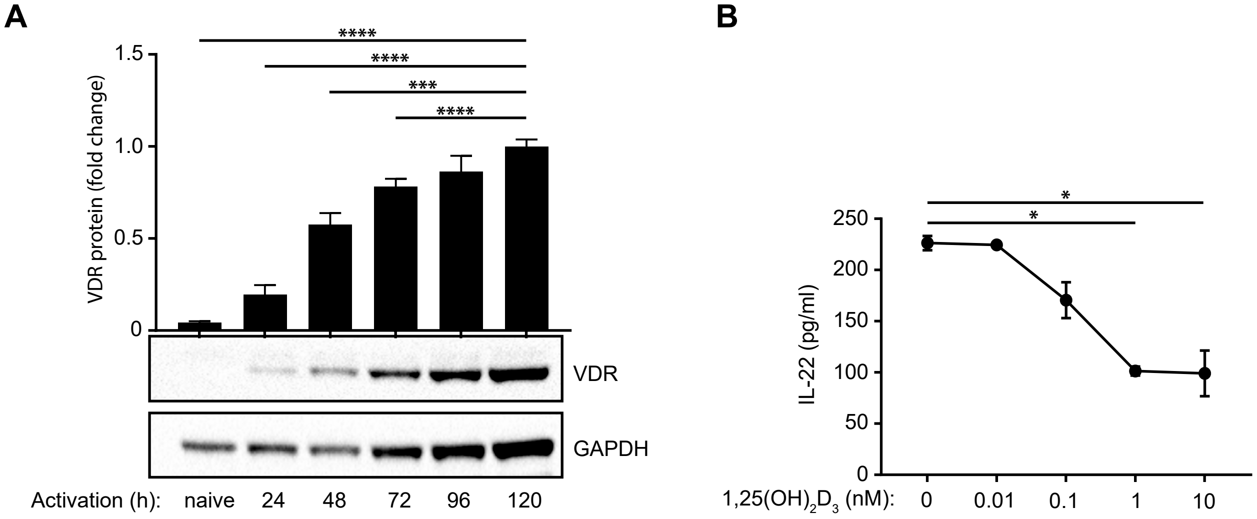

SUPPLEMENTARY FIGURE 3. (A) Western blotting analysis of VDR expression in naïve CD4$^+$ T cells stimulated with allogeneic DC in Th22 medium for the time indicated. The upper panel gives the density of the VDR normalized to the average density of the VDR bands at 120 h. The lower panel shows one representative Western blotting analysis of VDR and GAPDH (loading control). Data were obtained from three independent experiments with two donors in each experiment. **(B)** IL-22 in the supernatants of naïve CD4$^+$ T cells stimulated with Dynabeads Human T-activator CD3/CD28 in Th22 medium for 96 h. Data were obtained from one experiment with two donors.
