# Supplementary material for: Vitamin D Inhibits IL-22 Production Through a Repressive Vitamin D Response Element in the il22 Promoter
Source: Front Immunol. 2021 Aug 2;12:715059. doi: 10.3389/fimmu.2021.715059 (PMC8366496; doi:10.3389/fimmu.2021.715059)
Supplement: Supplementary file 4 [file Image_4.pdf]

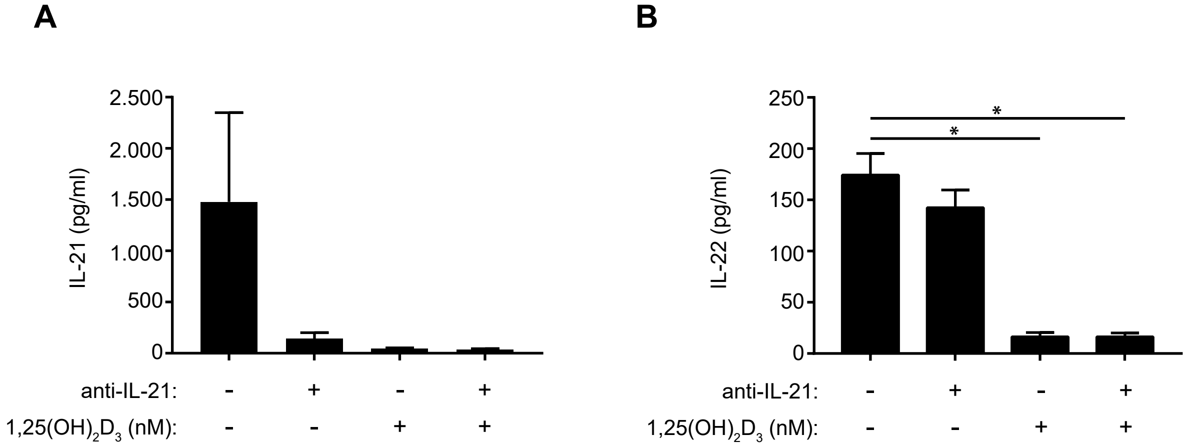

SUPPLEMENTARY FIGURE 4. **(A)** IL-21 and **(B)** IL-22 concentrations in the supernatant of T cells stimulated with Dynabeads Human T activator CD3/CD28 in X-VIVO 15 medium for 96 h in the absence or presence of 4 µg/ml neutralizing anti-IL-21 antibodies and 10 nM $1,25(OH)_2D_3$. Data were obtained from one experiment with three donors.
