# Supplementary material for: Vitamin D Inhibits IL-22 Production Through a Repressive Vitamin D Response Element in the il22 Promoter
Source: Front Immunol. 2021 Aug 2;12:715059. doi: 10.3389/fimmu.2021.715059 (PMC8366496; doi:10.3389/fimmu.2021.715059)
Supplement: Supplementary file 5 [file Image_5.pdf]

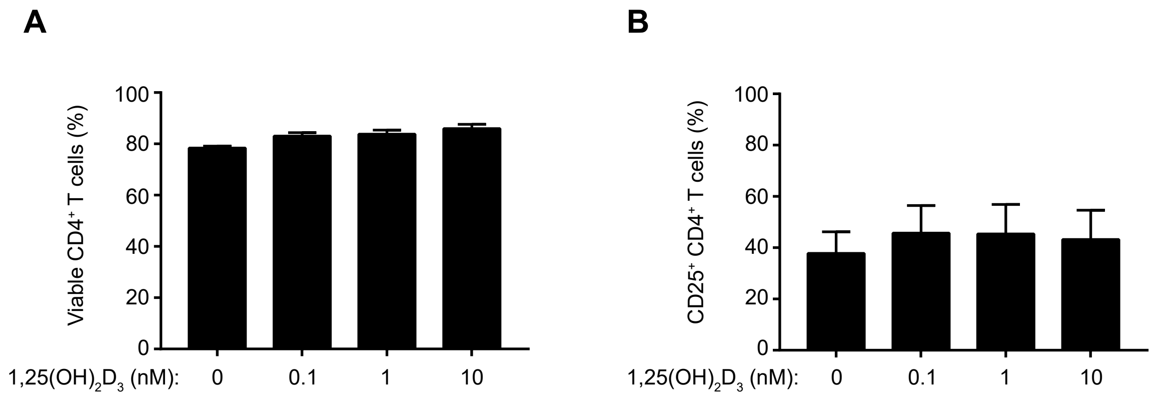

SUPPLEMENTARY FIGURE 5. (**A**) Cell viability and (**B**) expression of the activation marker CD25 on CD4$^+$ T cells stimulated with allogeneic DC in Th22 medium for 96 h in the presence of the indicated concentrations of 1,25$(OH)_2D_3$. Data were obtained from three independent experiments with five donors.
